# Combating Antimicrobial Resistance in Africa: A Call for Multidimensional Strategies to a Growing Crisis

**DOI:** 10.4314/ejhs.v34i4.1

**Published:** 2024-07

**Authors:** Esayas Kebede Gudina, Mulatu Gashaw, Tafese Beyene Tufa

**Affiliations:** 1 Department of Internal Medicine, Jimma University, Jimma, Ethiopia; 2 Jimma University Clinical Trial Unity, Jimma, Ethiopia; 3 School of Medical Laboratory Sciences, Jimma University, Jimma, Ethiopia; 4 CIH^LMU^ Center for International Health, Ludwig Maximilians Universität München, Munich, Germany; 5 College of Health Sciences, Arsi University, Asella, Ethiopia; 6 Hirsch Institute of Tropical Medicine, Asella, Ethiopia; 7 Department of Gastroenterology, Hepatology and Infectious Diseases, University Hospital and Heinrich Heine University, Düsseldorf, Germany

Antimicrobial resistance (AMR) is a critical global health threat, marked by microorganisms' ability to withstand antimicrobial treatments, making standard therapies ineffective and leading to persistent infections, increased morbidity, and mortality. The emergence of multidrug-resistant Gram-negative bacteria, in particular, is posing a significant challenge, and contributing to a high mortality globally (1).

Africa has the highest mortality rate attributable to AMR compared to other parts of the world (2). AMR in Africa is an escalating public health crisis driven by the overuse and misuse of antibiotics, inadequate healthcare infrastructure, and the widespread availability of substandard medications. Socio-economic disparities, limited access to diagnostic facilities, and weak regulatory frameworks further exacerbate the prevalence of AMR, leading to unregulated antibiotic consumption and the spread of resistant pathogens (3).

Traditional practices, such as self-medication, sharing of antibiotics, using of conventional medicines, excessive use of antibiotics in animals, unauthorized use of chemicals, and release of untreated wastes to the environment further complicate the AMR landscape (3). These behaviours contribute to the proliferation and persistence of multidrug-resistant organisms, complicating efforts to control and mitigate AMR. Moreover, healthcare systems in many African countries are inadequately equipped to manage resistant infections, resulting in heightened morbidity, mortality, and economic burden (4).

Addressing AMR in Africa requires a multifaceted approach, including enhanced surveillance systems, robust antimicrobial stewardship programs, and improved healthcare infrastructure (3). A “One Health” approach should be implemented, considering the influence of clinical and agricultural activities, vectors, and environmental hygiene on the spread of AMR in Africa (3-5). Effective policy implementation, regional and international collaborations, increased investment in research and development, and public awareness campaigns are crucial (1, 5).

Therefore, coordinated efforts are urgently needed to combat AMR and ensure the sustainability of effective antimicrobial therapies in Africa, emphasizing the importance of a holistic strategy that integrates scientific, regulatory, and community-based approaches. Failure to address AMR in Africa could reverse decades of progress in health outcomes and economic development, precipitating a crisis with far-reaching global implications.
